# Requirements for Admission to University of Michigan Dental Department

**Published:** 1891-03

**Authors:** 


### EDITORIAL.

### Requirements for Admission to University of Michigan—Dental Department.

The committee appointed by the senate of the University of Michigan to submit a schedule of minimum requirements for admission to the different departments of the University, also to propose some uniform plan of discipline throughout the University, held a meeting in the president's room January 11th and agreed upon the following schedule of admission, which should apply to all students entering any department of the University, with the exception of those students who are not candidates for a degree.

1. Matriculates in any of the regular courses in the literary department, graduates of literary colleges of good standing, graduates of schools approved as diploma schools by the literary department and of other high schools of equal standing, will be admitted without examination to any of the professional schools on the presentation of proper evidence to the secretary of the faculty.

2. For all others, requirements for admission shall be as follows:

(*a*) English Language: (1) A gramatical and retorical analysis of short selections in prose and poetry. The rhetorical analysis will be confined chiefly to the meaning and forms of words, sentential structure, paragraphing and figures of speech. (2) An essay of not less than two pages, (foolscap,) correct in spelling, punctuation, capital letters, sentential structure, paragraphing and figures of speech.

(*b*) Mathematical: *Arithmetic*, fundamental rules, fractions (common and decimal), denominate numbers, percentage, proportion, involution and evolution, and the metric system of weights and measures. *Algebra*, fundamental rules, portions, equations of the first degree, containing two or more unknown quantities.

*Geometry*, plane.

(*c*) Natural Philosophy: An amount represented by the study with experimental illustration of Avery's natural philosophy or Gage's elements of physics.

(*d*) Botany: The elements of vegetable anatomy and physiology as given in Gray's lessons.

(*e*) Zoology: Packard's zoology.

(*f*) Physiology: Martin's human body, briefer course.

(*g*) History: General history, Truman's general sketch of European history, Myers' general history or Swinton's outlines. Higginson's or Johnston's United States.

(*h*) Latin: Jones' first latin book or Harkness' latin reader, or an equivolent in any other book.

It is proposed that the above requirements be exacted of students entering the University after July, 1892.

The committee has ordered its secretary to refer this schedule to the various faculties and respectfully asks that the same be approved by each faculty.

In regard to discipline, the committee recommend the following:

1. That any student who has been convicted of any offense by the civil authorities, shall be, because of this conviction, considered as suspended from the privileges of the University.

2. That any student who shall, by any misconduct, forfeit his privilege in any department of the University, shall therefore be ineligible to membership in any department of the University.

